# Higher Urine Exosomal miR-193a Is Associated With a Higher Probability of Primary Focal Segmental Glomerulosclerosis and an Increased Risk of Poor Prognosis Among Children With Nephrotic Syndrome

**DOI:** 10.3389/fcell.2021.727370

**Published:** 2021-10-11

**Authors:** Lixia Wang, Jie Wang, Zhimin Wang, Jianhua Zhou, Yu Zhang

**Affiliations:** ^1^Department of Pediatrics, Tongji Hospital, Tongji Medical College, Huazhong University of Science and Technology, Wuhan, China; ^2^Department of Neonatology, Maternal and Child Health Hospital of Hubei Province, Tongji Medical College, Huazhong University of Science and Technology, Wuhan, China

**Keywords:** exosomes, microRNA-193a, focal segmental glomerulosclerosis, nephrotic syndrome, podocyte

## Abstract

**Background:** In children, focal segmental glomerulosclerosis (FSGS) is one of the most common primary glomerular diseases leading to end-stage renal disease. Exosomes facilitate communication between cells by transporting proteins and microRNAs. We aimed to investigate the utility of urine exosomal miR-193a for diagnosis and prognosis estimation among patients with primary FSGS, and preliminarily explore the regulation mechanism of exosome secretion from podocytes.

**Methods:** Specimens of urine were obtained from patients with primary FSGS, minimal change nephropathy (MCN) and IgA nephropathy (IgAN), followed by exosome isolation. We quantified urine exosomal miR-193a based on quantitative reverse transcription-polymerase chain reaction, and evaluated its applicability using area-under-receiver-operating-characteristics curves (AUROCs). The semiquantitative glomerulosclerosis index (GSI) was used to evaluate the degree of glomerulosclerosis according to the method of Raij et al. We further used FAM-labeled miR-193a-5p to examine exosome shuttling using confocal microscopy for visualization, and explored the regulation mechanism of exosomes release from podocytes using Fluo-3AM dye.

**Results:** Urine exosomal miR-193a levels were significantly higher in patients with primary FSGS than those with MCN and IgAN. The AUROCs for discriminating between primary FSGS and MCN or IgAN were 0.85 and 0.821, respectively. Urine exosomal miR-193a levels positively correlated with GSI in patients with primary FSGS. We further found that kidney tissues from these patients had increased CD63 expression involving podocytes in non-sclerotic tufts. Exosomes from cultured podocytes could transport miR-193a-5p to recipient cells, potentially through a calcium-dependent release mechanism.

**Conclusion:** Urine exosomal miR-193a might be harnessed as a non-invasive marker for diagnosis and outcome assessment among patients with primary FSGS. Exosomes were potential vehicles for miRNAs shuttling between podocytes, and released from podocytes in a calcium-dependent manner.

## Introduction

Primary focal segmental glomerulosclerosis (FSGS) is a frequent cause of nephrotic syndrome with a treatment-refractory course, and can be progressive. Having primary FSGS is associated with a 50% probability of developing end-stage renal disease (ESRD) after a mean duration of 5 to 8 years since biopsy, especially among patients not responsive to therapeutics or who were untreated (Korbet, 1999). Focal segmental glomerulosclerosis frequently recurs even after a successful renal transplantation, affecting up to 30% and 50% of adults and children, respectively (Vinai et al., 2010). From this perspective, earlier diagnosis and a timely treatment become an important goal if we want to improve patients’ long-term outcomes. The golden standard for diagnosing and classifying FSGS currently remains renal biopsy, whose disadvantages include sampling insufficiency leading to missing glomerular abnormalities. It is not uncommon to have difficulty distinguishing findings between early FSGS and minimal change disease. In addition, the invasiveness of renal biopsy and its association with several adverse events including bleeding and infections is also of concern. Finally, serial renal biopsy for dynamically monitoring disease course is deemed impractical from the clinical ground. Consequently, we are in urgent need of reliable non-invasive biomarkers to facilitate a prompt diagnosis and prognosis estimation for patients with FSGS.

MicroRNAs (miRNAs) are promising disease biomarkers owing to their disease-specificity and biological stability in different kinds of body fluid. Cumulative clinical and experimental reports have shown that miRNAs play an important role in the pathophysiology of renal diseases (Chung and Lan, 2015; Bhatt et al., 2016; Denby and Baker, 2016; Ledeganck et al., 2019). Gebeshuber et al. revealed that mice with transgenic miR-193a expressions developed FSGS rapidly, presenting as the effacement of podocyte foot processes extensively. In their study, an up-regulation of miR-193a levels could be observed in glomeruli isolated from adults with FSGS (Gebeshuber et al., 2013). Later, another group found that miR-193a mediated the switch between the parietal epithelia phenotype and that of podocytes (Kietzmann et al., 2015). Furthermore, patients with FSGS had significantly higher plasma miR-193a levels than healthy controls (Zhang C. et al., 2015). Although increased expression of miR-193a has been described in patients with FSGS, it remains unclear whether miR-193a contributes to glomerulosclerosis or whether miR-193a can be harnessed for early diagnosis and outcome assessment.

Exosomes are a class of extracellular membrane-bound vesicles released from host cells by fusion of the multivesicular body with the cell membrane. Exosomes can be secreted by every epithelial cell type lining the urinary tract system in humans (Pisitkun et al., 2004; Morrison et al., 2016). Their sizes are 30-100 nm and they harbor precursor miRNAs, mature miRNAs, mRNAs, single- and double-stranded DNA (ssDNA and dsDNA), mitochondrial DNA (mtDNA), and proteins within (Morrison et al., 2016). Exosomes are capable of acting in a paracrine manner by transmitting messages not only to nearby cells but also to other organs body-wide. Upon stimulation, transcriptomic changes within cells may modulate the assortment of miRNAs into exosomes (Squadrito et al., 2014). Besides, exosomes have lipid bilayers and can protect their cargoes from the degradation of RNase (Miranda et al., 2010; Cheng et al., 2014). Consequently, exosomes may serve as a potential source for uncovering new biomarkers. Exosomes can be obtained non-invasively, which is another advantage. Ever since the first report of urine exosome isolation in 2004, subsequent studies have tried to test the utility of urine exosomes as sources of biomarkers discovery in patients with different diseases including renal, urogenital and others (Miranda et al., 2010; Erdbrügger and Le, 2016). Along with the above rationale, we attempted to investigate whether urine exosomal miR-193a might be a potential biomarker for achieving an early diagnosis of primary FSGS and for outcome assessment. In addition, we performed some pilot experiments regarding the mechanisms through which exosomes were secreted by podocytes.

## Materials and Methods

### Patient Recruitment and Sampling

We recruited patients with primary FSGS (*n* = 8), IgA nephropathy (IgAN, *n* = 7), minimal change nephropathy (MCN) (*n* = 5), and healthy individuals (*n* = 5). The diagnosis of primary FSGS and IgAN was made based on input from expert pediatric nephrologists and pathologists who reviewed patients’ medical history, physical examination results, laboratory data, whole exome sequencing data and renal biopsy findings. The diagnosis of MCN was achieved based on clinical manifestation and laboratory data only, because children with MCN usually have no renal biopsy as most of these patients are steroid-sensitive. Exclusion criteria of this study included genetic testing-proven genetic FSGS, secondary FSGS, urinary tract infection, those with a positive family history of kidney diseases, congenital anomalies of the kidney and urinary tract, secondary nephrotic syndrome, those with diabetes, hypertension, with non-nephrotic proteinuria, those with an estimated glomerular filtration rate (eGFR) lower than 90 mL/min/1.73 m^2^ (using the Schwartz formula), and those receiving immunosuppressive treatments. Healthy controls were selected based on the following criteria: being free of any systemic and non-systemic diseases according to patients’ history and routine laboratory findings. To avoid selection bias, healthy controls and patients with different glomerulopathy were enrolled consecutively from January 2015 to July 2016.

The protocol of this study was approved by the Ethical Committee of Tongji Hospital, and written informed consent was obtained from all participants’ parents.

### Urine Sample Processing and Exosome Isolation

We obtained renal tissue specimens from renal biopsy prior treatment and collected morning urine samples from study patients at the day before biopsy. Ten milli-liters of first-time urine in the morning were centrifuged for 15 min at 3,000 g at 4°C for removing cells and/or debris. The remaining supernatants were stored at −80°C, followed by exosome isolation using the ExoQuick Exosome Precipitation Solution (System Biosciences, United States), according to the manufacturer’s instruction. This was achieved through incubating supernatant with an equal volume of ExoQuick-TC Exosome Precipitation Solution for 12 h at 4°C. Samples were subsequently centrifuged at 1,500 g for 30 min at 4°C. After discarding the residual supernatant, we collected exosome pellets, resuspended them in nuclease-free water of one-tenth of the original volume, and stored specimens at −80°C until the next step.

### Transmission Electron Microscopy Procedure

We fixed exosome-containing pellets in 100 μL glutaraldehyde and mounted them onto formvar/carbon-coated cooper grids. We removed excess fluid using a piece of dry filter paper. The loaded grids were transferred and stained with 0.75% uranyl formate for 30 s. Subsequently we dried them with filter paper and imaged the samples using a transmission electron microscopy (FEI Tecnai 20, Philips, United States), with sizes of exosomes measured by image.

### Western Blot Analysis

During western blotting, we loaded proteins on 10% sodium dodecyl sulfate polyacrylamide gel electrophoresis (SDS-PAGE) gels and transferred them to polyvinylidene fluoride (PVDF) membranes. Blots were then incubated with primary antibodies against TSG101 or HSP70 (dilution 1:1000, Abcam, United States), and later with horseradish peroxidase (HRP)-conjugated secondary antibody (dilution 1:10000, Santa Cruz Biotechnology, United States). We used Clarity^TM^ Western ECL substate kit (Bio-Rad, United States) to develop the blots and measured the intensity of bands using densitometric scanning based on the ImageJ software.

### Exosome RNA Extraction and Quantitation of miR-193a

We subjected 200 μL of resuspended exosome pellets to RNA extraction, using a Total Exosome RNA & Protein Isolation Kit (Applied Biosystems, United States), in compliance with the manufacturer’s protocol. We measured RNA concentration and purity based on the relative absorbance ratio at 260/280 (Nanodrop 2000, Thermo, United States). Subsequently, 10 ng of total RNA was reverse transcribed to cDNA using a miRNA cDNA Synthesis Kit (ABM Inc., Canada), followed by quantitative polymerase chain reaction (qPCR) using EvaGreen qPCR Master mix (ABM Inc., Canada) for miRNAs. We obtained primer mixes of RNU6 and has-miR-193a-5p from Genecopoeia lnc, and performed qPCR using the following condition: cycling initially at 95°C for 10 min, then at 95°C for 10 s, 63°C for 15 s, and finally at 72°C for 32 s, with a 7500 Fast Real-Time PCR System (Applied Biosystems, United States). We analyzed qPCR results using the 7500 System SDS software (Applied Biosystems, United States), and normalized the relative expression values of exosomal miR-193a to RNU6 using the 2^–ΔΔCt^ method.

### Histological and Immunohistochemical Studies

We fixed kidney tissues in neutral formalin, dehydrated and embedded them in paraffin, and cut the specimens in 2-μm-thick sections using a microtome. Sections were stained with hematoxylin & eosin (H&E), periodic acid-Schiff (PAS), and Masson’s Trichrome as general histological examinations. For immunohistochemical staining, we incubated the sections with rabbit anti-human CD63 antibodies (1:200, Wuhan Boster Biological Technology Co., Ltd, China), and then dewaxed, rehydrated, retrieved antigen, inactivated sections with 3% H_2_O_2_, and blocked non-specific binding with 10% goat sera. Negative control sections were also incubated with the antibody dilution vehicle [0.01 M phosphate-buffered saline (PBS) containing 0.3% Triton X-100]. We added secondary antibodies to the sections using the EnVision kit (DAKO, Denmark) after an overnight incubation at 4°C. After DAB developed, sections underwent gradient dehydration, transparency, and mounting. We observed sections under a light microscope (Olympus, Japan), and analyzed images by the Image Pro Plus 6.0 software.

### Calculating Glomerular Sclerosis Index

We measured the severity of glomerular sclerosis by a standard semiquantitative analysis, Glomerular Sclerosis Index (GSI), introduced by Raij et al. (1984). Briefly, at least 20 glomeruli in each specimen were assigned scores from 0, 1, 2, 3, to 4 if the glomeruli were normal, had less than 25, 25 to 50, 50, to 75, or more than 75% sclerosed area, respectively. We calculated GSI of each patient based on the following formula: N1 × 1 + N2 × 2 + N3 × 3 + N4 × 4)/n, in which N1, N2, N3, and N4 represented the numbers of glomeruli having grades 1, 2, 3, or 4, respectively, while n was the number of glomeruli assessed. Two independent observers (J.W. and Z.W.) blind to the experiment performed morphological analyses.

### Cell Cultures and the Isolation of Exosomes

We obtained immortalized human podocyte cells (AB8/13) from Dr. Kang YL (Shanghai Children’s Hospital, China), and cells were cultured in RPMI 1640 medium with 10% fetal bovine serum (Life Technologies, United States), 100 U/mL penicillin and 100 μg/mL streptomycin (Sigma-Aldrich, United States). We propagated the podocytes at 33°C and treated cells with Insulin-Transferrin-Selenium (Life Technologies, United States) to maintain their proliferative ability. Once reaching 50–60% confluence, cells were harvested, washed and cultured in a type I collagen-coated plate at 37°C for at least 10 days to allow differentiation. The differentiated cells were used for the following experiments.

To isolate exosomes, we collected medium followed by sequential centrifugation at 300 g for 10 min, 3,000 g for 15 min, and then at 10,000 g for 70 min at 4°C firstly. We further treated the supernatant with ExoQuick-TC for final exosomes isolation. Pellets containing exosomes were dissolved in 500 μL of 1 × PBS.

### Determining the Levels of Cytosolic [Ca^2+^]i by the Fluo-3AM Fluorescent Dye

This method has been described by Lencesova et al. (2013). In brief, we plated podocytes on a 24-well plate at the density of 4 × 10^4^. After 24 h of treatment, cells were washed with 1 mL of serum-free medium and we added 2 μM Fluo-3AM [4-(6-acetoxymethoxy-2, 7-dichloro-3-oxo-9-xanthenyl)-4′-methyl-2, 2′(ethylendioxy) dianiline-N,N,N′,N′-tetraacetic acid tetrakis (acetoxy methyl)ester] (Sigma-Aldrich, United States) with 1 mM probenecid (Sigma-Aldrich, United States) dissolved in serum-free medium, for 30 min at 37°C with the condition of 5% CO_2_ in darkness. Later, we washed cells with 1 mL of serum-free medium, and measured fluorescence using a flow cytometer at λ_ex_ 506 nm and λ_em_ 526 nm. We calculated results based on the mean fluorescence intensity. To chelate Ca^2+^ present in culture media, cells were incubated for several hours in the presence of 1.5 mM EGTA. BAPTA-AM was used to chelate intracellular Ca^2+^.

### Quantitation of Released Exosomes

We quantified the levels of exosomes through measuring acetylcholinesterase (AChE) activity, an enzyme specifically directed to these vesicles (Savina et al., 2002). In short, we suspended 25 μL of isolated exosomes in 100 μL of phosphate buffer and incubated them with 1.25 mM acetylthiocholine and 0.1 mM 5,5′-dithiobis (2-nitrobenzoic acid), achieving 1 mL as the final volume, in cuvettes at 37°C. We followed changes in absorbance at 412 nm continuously, and collected the enzymatic activity at 20 min after incubation as the results.

### Statistical Analysis

We described data as median (if non-normally distributed data) or mean ± standard deviation (if normally distributed data), and our experimental results were obtained from triplicate experiments. We compared data between groups using Kruskal Wallis test (if non-parametric variables) or Fisher’s least significant difference (LSD) test (if parametric variables). We evaluated the diagnostic performance of urine exosomal miR-193a for primary FSGS by calculating the sensitivity and specificity using the receiver-operating-characteristic (ROC) curves. We used one-dimensional regression to analyze the correlations between two parameters. A *P* value lower than 0.05 was considered statistically significant. We used the SPSS software package (version 13.0) for statistical analysis and created charts using the GraphPad Prism version 6 for Windows.

## Results

### The Characteristics of Participants

We summarize baseline demographic and clinical features of study participants in Table 1. There were no significant differences in gender, race and eGFR between the primary FSGS group and other groups. Similarly, there were no significant differences in serum albumin, proteinuria and the doses of steroid between the FSGS, MCN, and IgAN groups. Significant differences existed in age between those of the MCN group and those of the other groups, because MCN is the most common origin of nephrotic syndrome among children aged between 1 and 6 years.

**TABLE 1:** Clinical and laboratory characteristics of study subjects.

	FSGS (*n* = 8)	MCN (*n* = 5)	IgAN (*n* = 7)	Control (*n* = 5)
***Demographic***				
Age, year	8.2 ± 1.0*	2.8 ± 0.3	10.2 ± 0.98	5.9 ± 1.7
Sex, male/female	6/2	2/3	4/3	3/2
Race/ethnicity, n (%)				
*Han*	100%	100%	100%	100%
***Laboratory parameters***				
eGFR (mL/min)	> 90	> 90	> 90	> 90
Proteinuria (g/24h)	3.587 ± 1.484^#^	3.242 ± 1.631	3.590 ± 0.680	0
Serum albumin (g/L)	22.7 ± 3.5	20.2 ± 2.2	28.7 ± 3.4	40.5 ± 1.5
***Treatment***				
Dose of steroid	2 mg/kg/d	2 mg/kg/d	2 mg/kg/d	n.d.

Values are means ± SE. eGFR, estimated glomerular filtration rate; n.d., not determinate. *P*-value refers to the comparison of the FSGS with healthy controls and the values in bracket referred to the comparison with MCN: Mann-Whitney *U* Test or Pearson χ^2^ test. * FSGS *vs.* MCN *P* < 0.05, # FSGS *vs.* healthy controls *P* < 0.05.

### Characterizing Urine Exosomes

**FIGURE 1:**
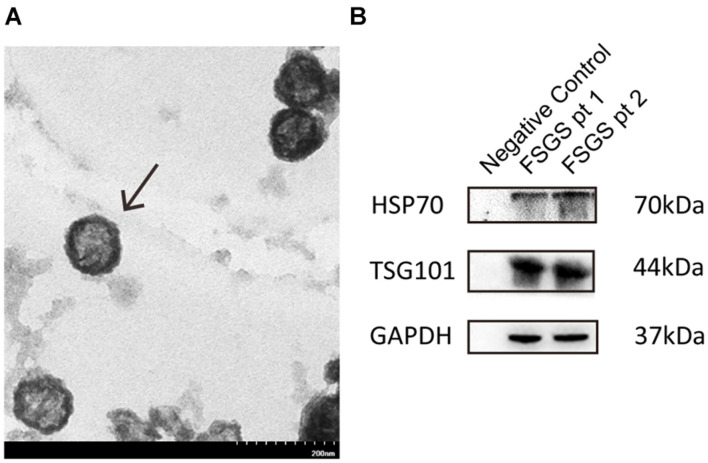
Identification and characterization of exosomes. **(A)** Urine exosomes were isolated from urine by the ExoQuick exosome precipitation solution. Urine vesicles, showing the characteristic exosomal cup-shape and size, are shown in the representative electron micrographs; scale bar = 200 nm. **(B)** Expression of exosomal markers (HSP70, CD9) was assessed by immunoblotting in total protein extracts from isolated urinary exosomes.

We examined urine exosomes using an electron microscopy, and found vesicles with an average of 65.44 ± 20.1 nm diameters and a typical spherical shape (Figure 1A). Results from Western blot showed that exosomal markers including TSG101 and HSP70 could be detected at molecular weight levels of 44 and 70 kDa, respectively (Figure 1B). The findings of our isolated vesicles were compatible with the typical features of exosomes (Zhang J. et al., 2015), and the quality could be satisfactory and suitable for subsequent experiments.

### Urine Exosomal miR-193a in Patients With Primary Focal Segmental Glomerulosclerosis and the Control Groups

**FIGURE 2:**
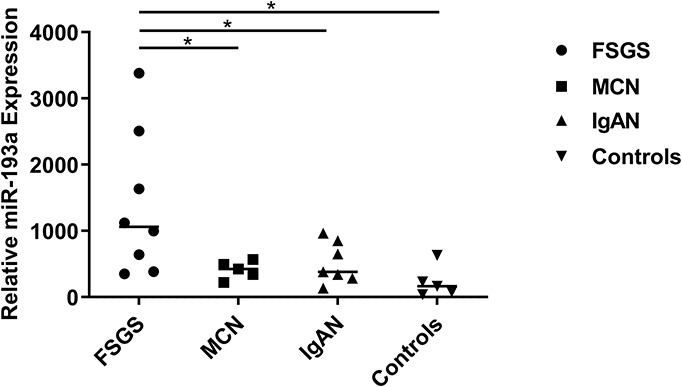
Relative expression levels of urine exosomal miR-193a between patients with FSGS patients and controls. Relative expressions were shown which was calculated with the equation 2^–*delta Ct*^, normalized for RNU6 and relative to the control average. Scatter plot (middle line: median). *P < 0.05.

As shown in Figure 2, the expression levels of urine exosomal miR-193a were significantly higher in the primary FSGS group than those of other groups (FSGS: 1060.4 *vs.* MCN: 423.7, IgAN: 381.9, Control: 163.6). These findings suggested that urine exosomes could potential be utilized for identifying miR-193a, a biomarker capable of differentiating primary FSGS from other glomerular diseases including MCN.

### Discriminative Power Using miR-193a for Identifying Primary Focal Segmental Glomerulosclerosis

**FIGURE 3:**
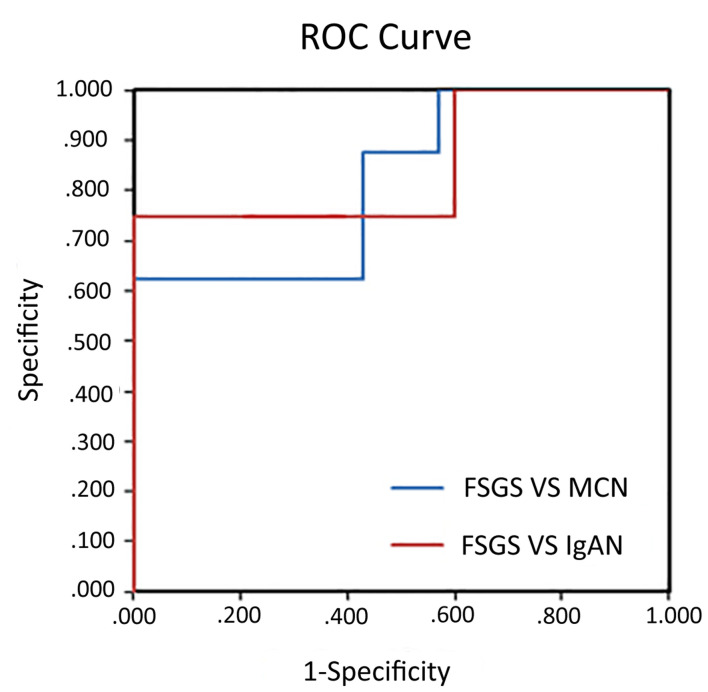
Diagnostic values of urine exosomal miR-193a levels in patients with primary FSGS. ROC curve analysis of miR-193a expression to discriminate between primary FSGS patients and MCN or IgAN patients. The areas under the ROC curve were 0.85 and 0.821, respectively.

To assess the diagnostic ability using urine exosomal miR-193a for primary FSGS, we used ROC curves for discriminating primary FSGS from other glomerular diseases. The areas under the ROC curve (AUCs) of using urine exosomal miR-193a for differentiating primary FSGS from MCN or IgAN were 0.85 and 0.821, respectively (Figure 3).

### Urine Exosomal miR-193a Expression Correlates Positively With Glomerular Sclerosis Index in Patients With Primary Focal Segmental Glomerulosclerosis

**FIGURE 4:**
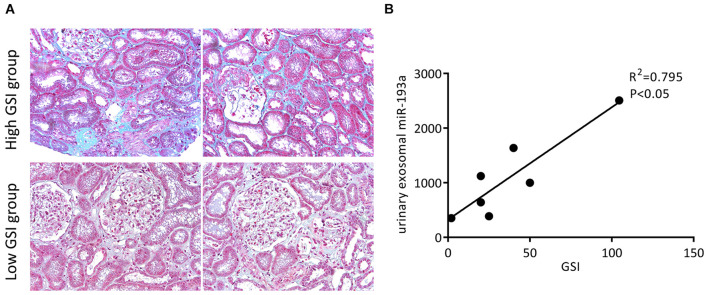
Prognostic values of urine exosomal miR-193a levels in patients with primary FSGS. **(A)** Representative photomicrographs of the renal biopsy with Masson’s trichrome staining (200×). **(B)** The correlation between the expression level of urine exosomal miR-193a with GSI in patients with primary FSGS.

We collected 8 renal tissue samples from patients with primary FSGS and calculated their GSI. Figure 4A shows the representative Masson trichrome staining results of renal sections from each group. Urine exosomal miR-193a expressions were positively correlated with the severity of glomerular sclerosis. Patients with a higher GSI had significantly higher levels of urine exosomal miR-193a compared to those with a low GSI (Figure 4B). Among the spectrum of renal pathological changes, only glomerular sclerosis was correlated with miR-193a expressions, while there was no correlation between the degree of tubular atrophy or interstitial fibrosis and miR-193a expressions.

### CD63 Expressions Increase Within Glomeruli From Patients With Primary Focal Segmental Glomerulosclerosis

**FIGURE 5:**
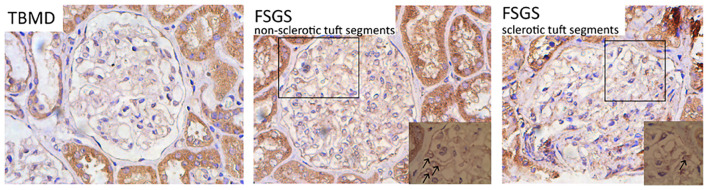
Representative micrographs show renal CD63 staining (IHC, 400×). The expression level of CD63 was significantly up-regulated in patients with FSGS compared with TBMD, which was mainly located in the cytoplasm of podocytes within non-sclerotic tuft segments (indicated by black arrows).

To examine whether urine exosomal miR-193a came from podocytes and whether it could be used as a biomarker for diagnosing primary FSGS, we evaluated CD63 expressions (exosome marker) in the renal tissue sections. We found that renal CD63 expressions were significantly up-regulated in patients with FSGS compared to those with thin basement membrane disease (TBMD). In the FSGS cases, renal CD63 expressions were mainly located in podocyte cytoplasm within non-sclerotic tuft segments (Figure 5). This finding suggested that the formation of multivesicular bodies increased within the podocytes in patients with primary FSGS.

### Podocyte-Derived Exosomes Can Deliver miR-193a to Recipient Cells *in vitro*

**FIGURE 6:**
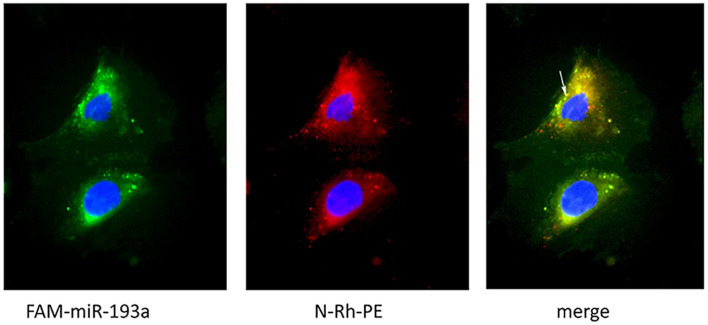
Visualization of exosomes shuttling FAM-labeled miR-193a-5p to recipient podocytes. Recipient podocytes were incubated with donor podocytes-derived exosomes (10 μg/ml). Yellow fluorescent “specs” (indicated by white arrow) represent N-Rh-PE labeled exosomes containing FAM-miR-193a-5p taken up by the recipient podocytes.

We further visualized the transfer of labeled miRNAs between podocytes *in vitro*. We first transfected podocytes with FAM-labeled miR-193a-5p. Twenty-four hours later, we used a fluorescent phosphatidyl ethanolamine analog, N-Rh-PE [1,2-dipalmitoyl-sn-glycero-3-phosphoethanolamine-N-(lissamine rhodamine B sulfonyl)], to label exosomes. We collected culture medium 1 day later for isolating exosomes. An independent set of recipient podocytes was then incubated with 10 μg of isolated exosomes for 24 h. We showed that N-Rh-PE-labeled exosomes were endocytosed, and FAM-labeled miR-193a-5p was localized in exosomes, as were visualized by a fluorescence microscopy (Figure 6). Our findings indicated that podocyte-derived exosomes could transport miRNAs to recipient cells.

### A Calcium-Dependent Mechanism Is Involved in Exosome Release

**FIGURE 7:**
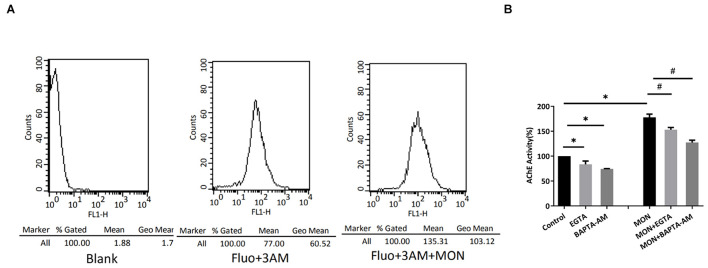
Monensin stimulates exosome release by a calcium-dependent mechanism. **(A)** Podocytes were loaded with Fluo-3AM, and intracellular Ca^2+^ concentration was measured. Shown are changes in Ca^2+^ concentration elicited by 7 μM MON. Data are representative of at least three independent experiments. **(B)** Podocytes were incubated for 7 h in the presence of 1.5 mM EGTA, 30 μM BAPTA-AM, 7 μM MON, or the combination of 1.5 mM EGTA/7 μM MON (MON + EGTA) or 30 μM BAPTA-AM/7 μM MON (MON + BAPTA-AM). The secreted exosomes were collected and quantitated by measuring the AChE activity. ^∗^ significantly different from the control, *P* < 0.05. # significantly different from the MON-treated cells, *P* < 0.05.

Intracellular Ca^2+^ rise is found to be necessary for the induction of regulated secretion in most cell types (Gerber and Südhof, 2002; Wasle and Edwardson, 2002). During exocytosis under regulation, the membrane of secretory vesicle fuses with the plasma membrane precipitated by a tightly controlled Ca^2+^-triggered reaction. Because monensin (MON), a membrane-permeable Na^+^ ionophore known to induce Ca^2+^ entry through reversed activity of the Na^+^/Ca^2+^ exchanger (Wang et al., 1999), we tested the influence of MON on the release of exosomes from podocytes and observed whether Ca^2+^ was involved in this process. We harvested exosomes from culture media after 7 h of incubations with 7 μM MON and measured AChE activity. Monensin treatment was found to induce a marked increase in the amount of exosome released (Figure 7B). To assess whether the MON-induced exosome release was related to intracellular Ca^2+^ increase, we evaluated whether MON modified intracellular Ca^2+^ concentrations in cultured podocytes. We found a significant increased intensity of Fluo-3AM fluorescence (Figure 7A), suggesting that the increase in exosome release might be Ca^2+^ dependent. We also assessed whether the effect of MON effect could be prevented by Ca^2+^ chelators. Both EGTA and BAPTA-AM slightly decreased the basal levels of exosome release (Figure 7B). The MON-dependent increase was significantly abrogated by both Ca^2+^ chelators, indicating that Ca^2+^ present in culture media and also in intracellular space was required for exosome secretion from podocytes.

## Discussion

Focal segmental glomerulosclerosis (FSGS), a primary podocytopathy, is characterized histologically by extensive podocyte injury with segmental glomerular scarring. Podocytes, an important pathologic player, cover the outer layer of the glomerular basement membrane and assist in stabilizing glomerular architecture as well as function. Podocyte-origin exosomes have been shown to be detectable in urine from previous studies (Zhou et al., 2013; Hogan et al., 2014; Lv et al., 2014; Gámez-Valero et al., 2015). Similarly, we showed that patients with primary FSGS had podocytes with an increased exosome production if compared to those with TBMD. We also found massive quantities of detectable exosomes in the urine of patients with MCN and IgAN, suggesting that urine exosomes serve as potential sources of biomarkers for diagnosing glomerular diseases while sera or plasma may be inappropriate due to their containing exosomes of non-renal origin. Besides, functional cells can selectively incorporate miRNAs into exosomes followed by releasing them extracellularly, in response to multiple adverse stimulations (Zhang et al., 2010; Chen et al., 2012; Eirin et al., 2014; Nishida-Aoki and Ochiya, 2015). It is reasonable to hypothesize that urinary exosomal miRNAs could be used as a more excellent mirror to reflect the renal pathological change compared with miRNAs gained from whole urine sample. Unlike renal biopsy, the collection of urine exosomes is relatively non-invasive, convenient, and amenable to repeated performance. In addition, exosomes can protect their internal content from the degradation of RNases and proteinases. These biologic advantages suggest that using enriched urine exosomes as a source of biomarkers may increase the sensitivity, specificity and practicality of miRNA for predicting podocytopathy. So far, few reports addressed the utility of urine exosomal miRNAs as biomarker for diagnosing primary glomerular disorders. Lv et al. (2013) discovered that urine exosomal miR-29c was a biomarker correlating with renal function and fibrosis in patients with chronic kidney disease (CKD). Ramezani et al. (2015) reported that urine exosomal miR-1915 and miR-663 were downregulated while miR-155 was upregulated in patients with FSGS. We believe that more urine exosomal miRNAs as diagnostic biomarker are needed for patients with primary FSGS. Prior experiments showed that mice with transgenic miR-193a expressions developed FSGS (Gebeshuber et al., 2013), and we enrich their findings by showing the diagnostic and prognosis estimating ability of urine exosomal miR-193a for primary FSGS.

We used to establish a handy and reliable protocol for the isolation of exosomes from urine, and further showed that urine exosomal miR-193a significantly increased in patients with primary FSGS patients compared to those with MCN (Huang et al., 2017). In this study, we attempted to characterize the utility of urine exosomal miR-193a as a diagnostic marker for primary FSGS, through comparing their levels in those with primary FSGS to those of patients with other primary glomerulopathies including MCN and IgAN and those of healthy controls. We found that urine exosomal miR-193a levels were significantly higher in the primary FSGS group than those of other groups. Results of ROC analyses support the utility of urine exosomal miR-193a for discriminating primary FSGS from MCN and IgAN. Therefore, we believed that urine exosomal miR-193a might be a potential diagnostic biomarker for primary FSGS. In addition, urine exosomal miR-193a levels also correlated with GSI while not with tubular and interstitial lesions. Gebeshuber et al. (2013) revealed that miR-193a inhibited WT1 expressions, leading to the downregulation of WT1 targets including *PODXL* and *NPHS1*. This action exerts a catastrophic influence on the entire podocyte-stabilizing system. From this view, miR-193a plays a critical role in glomerular sclerosis development and urine exosomal miR-193a could be directly linked to the severity of podocyte injuries.

Matsusaka et al. (2011) established chimeric mice with 25–50% podocytes expressing immunotoxin LMB2 receptor hCD25, and the mice were subsequently stimulated to develop hCD25-positive podocytes-specific injuries through injecting recombinant immunotoxin LMB2. They demonstrated that, although LMB2 was rapidly metabolized with a half-life of 35 min, injuries to podocytes worsened over weeks, culminating in FSGS development 6 weeks after injection. Their findings clearly indicated that the expansion of segmental sclerosis involved gradual podocyte loss unrelated to the primary insult and were related to the autonomous propagation of podocyte-to-podocyte damages. Exosomes are purported as vital messengers participating in intercellular communication and exosomes secreted by one cell can interact with recipient cells, inducing adverse changes in recipient cells. According to findings of our study, significantly increased exosomes formation could be observed in podocytes from patients with primary FSGS compared to those from patients with TBMD. In order to identify whether exosomes could participate in the podocyte-to-podocyte transmission of damages, we conducted *in vitro* experiments to visualize the process of signal transmission by exosomes between podocytes. Using a confocal microscopy, we found that exosomes isolated from podocytes could deliver miR-193a to recipient podocytes. The induction of podocyte-secreted exosomes was calcium-dependent.

The main limitations in this study included the following: first, this study recruited relatively low number of patients, and future studies using a larger cohort will be necessary to validate our results. Second, we did not identify miRNA intrarenal expressions simultaneously. Third, our study is a cross-sectional one. Finally, the regulatory mechanisms of exosomes secretion from podocytes were not addressed in detail, and there are more to be explored.

## Conclusion

The present study identified two important findings that were different from previous reports, with regard to the exact pathophysiologic role of miR-193a in FSGS. First, our study might be one of the few pilot reports focusing on both the diagnostic and prognostic value of urine exosomal miR-193a for patients with primary FSGS. Secondly, we showed that *in vitro*, exosomes could transport miRNAs between podocytes in a calcium-dependent manner.

## Data Availability Statement

The original contributions presented in the study are included in the article/supplementary material, further inquiries can be directed to the corresponding author.

## Ethics Statement

The studies involving human participants were reviewed and approved by the Ethical Committee of Tongji Hospital. Written informed consent to participate in this study was provided by the participants’ legal guardian/next of kin.

## Author Contributions

YZ contributed to conception and design of the study. LW and JW completed the experiment and performed the statistical analysis. YZ wrote the first draft of the manuscript. All authors contributed to manuscript revision, read, and approved the submitted version.

## Conflict of Interest

The authors declare that the research was conducted in the absence of any commercial or financial relationships that could be construed as a potential conflict of interest.

## Publisher’s Note

All claims expressed in this article are solely those of the authors and do not necessarily represent those of their affiliated organizations, or those of the publisher, the editors and the reviewers. Any product that may be evaluated in this article, or claim that may be made by its manufacturer, is not guaranteed or endorsed by the publisher.
